# HBZ and its roles in HTLV-1 oncogenesis

**DOI:** 10.3389/fmicb.2012.00247

**Published:** 2012-07-09

**Authors:** Tiejun Zhao, Masao Matsuoka

**Affiliations:** ^1^Laboratory of Virus Control, Institute for Virus Research, Kyoto University,Kyoto, Japan; ^2^College of Chemistry and Life Sciences, Zhejiang Normal University,Jinhua, China

**Keywords:** HBZ, HTLV-1, Tax, viral oncogenesis, regulatory T cells

## Abstract

Human T-cell leukemia virus type 1 (HTLV-1) causes adult T-cell leukemia (ATL). The minus strand of HTLV-1 provirus encodes a bZIP protein donated as HTLV-1 bZIP factor (HBZ). Among the HTLV-1 regulatory and accessory genes, the *tax* and *HBZ* genes were thought to play critical roles in oncogenesis. However, HBZ is the only gene that remains intact and is consistently expressed in all ATL cases, while the tax gene is frequently inactivated by epigenetic modifications or deletion of the 5’LTR. *HBZ* gene promotes the proliferation of ATL cells through its mRNA form. Moreover, HBZ induces T-cell lymphoma and systemic inflammation *in vivo*. HBZ fulfills its functions mainly through regulating HTLV-1 5’LTR transcription and modulating a variety of cellular signaling pathways which are related with cell growth, immune response, and T-cell differentiation. Taken together, the multiple functions of HBZ render its predominant function in leukemogenesis of ATL.

## INTRODUCTION

**FIGURE 1:**
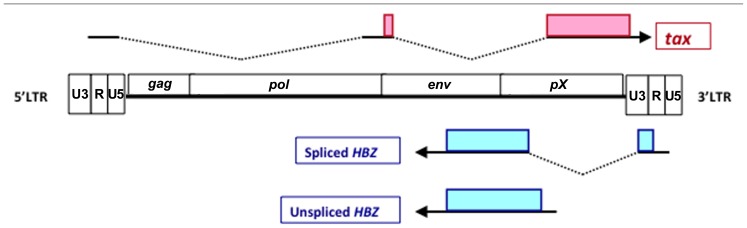
**Schematic diagram of spliced HBZ, unspliced HBZ and Tax.** Tax is encoded by the positive strand of HTLV-1 proviral genome. HBZ is located on the complementary strand and transcribed from 3′ LTR. Spliced and unspliced HBZ are showed. Shaded boxes represent their coding regions.

Human T-cell leukemia virus type 1 (HTLV-1) was the first retrovirus proven to be associated with human disease. The infection of HTLV-1 causes adult T-cell leukemia (ATL) and chronic inflammatory diseases including HTLV-1-associated myelopathy (HAM)/tropical spastic paraparesis (TSP; Uchiyama et al., 1977; Poiesz et al., 1980). HTLV-1 belongs to deltaretrovirus family, which include HTLV-2, HTLV-3, and HTLV-4; the simian T-cell lymphotropic viruses (STLV-1, STLV-2, and STLV-3) and the bovine leukemia virus (BLV; Matsuoka and Jeang, 2007). Like other retroviruses, the HTLV-1 proviral genome has structural genes, *gag*, *pol*, and *env*, flanked by long terminal repeat (LTR) at both ends (**Figure 1;** Matsuoka and Jeang, 2007). The characteristic of HTLV-1 proviral genome is the presence of pX region between env and 3′ LTR, and encoded several accessory genes, which include *tax*, *rex*, *p12*, *p21*, *p30*, *p13*, and *HTLV-1 bZIP factor* (*HBZ*; Gaudray et al., 2002; Nicot et al., 2005). HBZ was first identified as an antisense viral protein that associated with cAMP-response element binding protein-2 (CREB-2) in HTLV-1-infected cells (Gaudray et al., 2002). We found that the expression of HBZ is conserved in all ATL cells (Satou et al., 2006), and non-sense mutations of all HTLV-1 genes except the *HBZ* gene were generated byAPOBEC3G before integration of the provirus (Fan et al., 2010). In addition, the *HBZ* gene promotes the proliferation of ATL cells (Satou et al., 2006). Thus, the *HBZ* gene is believed to be essential for leukemogenesis. In this review, we discuss the updated understanding of HTLV-1 and focus on the roles of HBZ in HTLV-1 oncogenesis.

## STRUCTURE OF HBZ

The existence of a viral transcript encoded by the minus strand of the HTLV-1 provirus was reported in 1989 (Larocca et al., 1989). Thereafter, a viral protein that binds to CREB-2 was identified by yeast 2-hybrid screening system, and termed as HTLV-1 bZIP factor (HBZ; Gaudray et al., 2002). The 5′ RACE experiment identified the transcription start sites of HBZ were located in the U3 and R regions of the HTLV-1 3′ LTR (Satou et al., 2006). 5′ LTR of HTLV-1 was frequently lost and methylated in ATL cases, while 3′ LTR was not deleted and remained unmethylated (Matsuoka and Jeang, 2007). It may be one of the reasons why HBZ is consistently expressed in all ATL cells. Two transcripts of the *HBZ* gene have been revealed: a spliced form (s*HBZ*) and an unspliced form (us*HBZ*; **Figure 1).** The promoter of the spliced and unspliced HBZ transcripts was TATA-less and contained initiators and downstream promoter elements (Yoshida et al., 2008). Detailed analyses of the promoter of s*HBZ* gene showed that Sp1 sites were critical for its transcription. U3 region of LTR in the both end of HTLV-1 proviral genome contains Tax-response element (TRE), which acts as an enhancer of viral transcription. Tax and CREB can activate transcription of the *HBZ* gene through 3′ LTR, meanwhile, Tax regulated antisense promoter activity is influenced by the integration site. However, Tax induced 3′ LTR activation is not as significant as it did on the transcription of the HTLV-1 plus strand (Landry et al., 2009).

**FIGURE 2:**
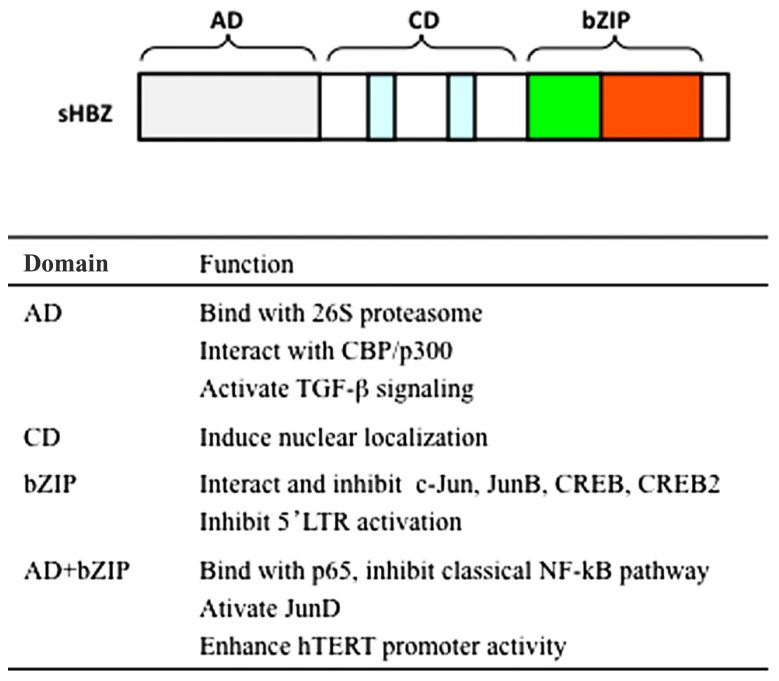
Schematic diagram of HBZ domains. HBZ has three domains: activation domain (AD), central domain (CD), and basic ZIP domain (bZIP). Functions of each domain are listed.

The spliced and unspliced *HBZ* genes are translated into a polypeptide of 206 and 209 amino acids, respectively. Both forms of HBZ contain three domains: N-terminal activation domain (AD), central domain (CD), and basic ZIP domain (bZIP) in the C-terminal (**Figure 2).** There are two LXXLL-like motifs located within the N-terminal AD domain of HBZ. Both are important for the binding to p300/CBP (Clerc et al., 2008). The HBZ protein is localized in the nucleus with a speckled pattern. Three nuclear localization signals (NLSs) were identified to two regions in the CD of HBZ and a basic region of the bZIP domain (Hivin et al., 2005). Moreover, the integrity of HBZ protein sequence was necessary for its accumulation in nuclear speckles. HBZ specifically distributed in the nucleus to heterochromatin, while it is not associated with heterochromatin (Hivin et al., 2005).

The only difference between the protein form of sHBZ and usHBZ are seven amino acids in their N-terminal, while it causes significant difference between these two proteins. The half-life of usHBZ protein is much shorter than that of sHBZ (Yoshida et al., 2008). In ATL cell, only sHBZ protein could be detected, since s*HBZ* gene is more predominant than us*HBZ* gene (Usui et al., 2008).

## TRANSCRIPTION OF THE *HBZ* GENE

The transcription of HTLV-1 viral gene is the symbol of HTLV-1 infection. Among all of the HTLV transcripts, the *tax* gene is thought to play critical role in leukemogenesis because of its pleiotropic functions. However, about 60% of ATL cases lost the tax expression by three mechanisms: (1) deletion of HTLV-1 5′ LTR, (2) hypemethylation of 5′ LTR, and (3) non-sense mutation, deletion, and insertion of tax gene (Matsuoka and Jeang, 2007). Moreover, the loss of Tax is believed to occur during the process of oncogenesis. It indicates that Tax expression is not always necessary for ATL. Unlike 5′ LTR, HTLV-1 3′ LTR is intact and remains unmethylated. The *HBZ* gene expression was conserved in all ATL cases. In addition, expression of the *HBZ* gene could be detected not only in ATL cells but also in HTLV-1-infected carriers, furthermore, its expression are well correlated with proviral load. It has been reported the correlation between the level of the *HBZ* gene and severity of HAM/TSP, indicating that HBZ might contribute to the development of HAM/TSP except for ATL (Saito et al., 2009).

The two forms of the *HBZ* gene are not equally expressed in ATL cells. The expression level of the s*HBZ* gene was found to be four times higher than that of the us*HBZ* gene transcript in HTLV-1 carriers and ATL cases.

In PBMCs from HTLV-1-infected rabbits revealed that the *tax* gene was expressed at the highest levels immediately after infection and progressively decreased, eventually stabilizing at low levels. However, *HBZ* was detectable at low levels early after infection, and slowly increased and stabilized. At 8 weeks post infection, the *HBZ* mRNA was expressed at the highest concentration of all mRNA measured (an average of ninefold more than *tax* mRNA; Li et al., 2009). Additionally, Suemori et al. (2009) reported that the expression levels of *HBZ* mRNA and HBZ protein in HTLV-1-infected cells were not parallel, suggest that the post transcriptional regulation of *HBZ* gene may differ among different cell types. However, other group detected the consistently high level of HBZ mRNA and protein in ATL- and HTLV-1-associated cell lines (Satou et al., 2006; Zhao et al., 2011). The physiological level of HBZ could regulate the cellular signaling pathway, which is critical for T-cell differentiation. The discrepancy between two independent studies may due to the different experiment condition and different HBZ antibody they used.

## THE BIOLOGICAL FUNCTION OF HBZ

### INHIBITION OF TAX-MEDIATED VIRAL TRANSCRIPTION

HBZ was firstly identified as a CREB-2 binding protein which could inhibit HTLV-1 transcription from 5′ LTR. Further study showed the association between HBZ and CREB (Lemasson et al., 2007). HBZ bZIP domain is responsible for its interacting with CREB-2/CREB. These interactions abolished the binding of CREB-2/CREB to the Tax-responsive element (TxRE) in the HTLV-1 5′ LTR and the cyclic AMP-response element (CRE) in cellular promoters, resulting in the suppression of Tax-mediated transcription from 5′ LTR (Gaudray et al., 2002; Lemasson et al., 2007). In addition, HBZ interacts with CBP/p300 co-activator and suppressed the transcription from the 5′ LTR by Tax (Clerc et al., 2008). Through two LXXLL-like motifs in the N-terminal AD, HBZ binds with CBP/p300 KIX domain, inhibiting the recruitment of CBP/p300 to the HTLV-1 promoter. Further study found that HBZ-AD interacts with the MLL binding surface of KIX domain (Cook et al., 2011).

### PROLIFERATION OF ATL CELLS

Stable expression of HBZ maintains the growth of Kit 225 cells (Satou et al., 2006). When knock down the expression of HBZ in ATL cell lines, MT-1 and TL-Om1, by siRNA, their proliferation was suppressed (Satou et al., 2006). It indicated that HBZ is essential for the continuous expansion of ATL cells. Furthermore, studies of the growth of T-cell lines transfected with the mutated *HBZ* genes showed that HBZ promotes T-cell proliferation in its mRNA form, whereas HBZ suppresses Tax-mediated viral transcription via protein form. Mutation analyses of the *HBZ* gene showed that *HBZ* RNA promotes cell proliferation by forming secondary stem-loop structures. *HBZ* RNA activates transcription of E2F1 and its target genes, and increases G1/S transition (Satou et al., 2006).

Spliced HBZ could induce the proliferation of ATL cells, while usHBZ could not (Yoshida et al., 2008). It suggests that the first exon of the sHBZ transcript is critical for its growth promoting activity. The first exon of HBZ corresponds to the Rex-responsive element (RxRE) of the HTLV-1 3′ LTR. It is known that Rex promotes the export of viral RNA with an RxRE region. Together, we speculate that s*HBZ* RNA form stem-loop structure, which different as us*HBZ* RNA, inducing the proliferation of ATL cells.

ATF3, an HBZ binding protein, was highly expressed in ATL cells. ATF3 expression has positive and negative effects on the proliferation and survival of ATL cells. HBZ impairs its negative effects, leaving ATF3 to promote proliferation of ATL cells via mechanisms including upregulation of CDC2 and cyclin E2. HBZ may take it as advantages to promote the cell survive (Hagiya et al., 2011).

### MODIFICATION OF AP-1 SIGNALING

Previous studies show that the interaction between bZIP proteins affects their cellular localization and modulates transcriptional activity. Activator protein-1 (AP-1) functions as a dimer comprised of bZIP proteins of Jun family (c-Jun, JunB, JunD), Fos family (c-Fos, FosB, Fra-1, Fra-2), and others. HBZ interacted with c-Jun/JunB and repressed c-Jun/JunB mediated signaling by abrogating its DNA-binding capacity. HBZ induced the degradation of c-Jun protein through a proteasome-dependent pathway, while it is in a ubiquitin-independent manner (Basbous et al., 2003; Matsumoto et al., 2005; Isono et al., 2008). In addition, HBZ inhibited AP-1 signaling by sequestrating c-Jun in nuclear bodies. On the contrary, HBZ can activate transcription mediated by JunD via forming heterodimers with JunD (Thebault et al., 2004). HBZ/JunD heterodimers interact with Sp1 transcription factors and enhanced the transcription of telomerase reverse transcriptase (hTERT; Thebault et al., 2004). Since telomerase activation is thought to be associated with tumor malignant, the induction of hTERT by HBZ may be implicated in the oncogenesis of ATL.

### SUPPRESSION OF THE CLASSIC NF-κB PATHWAY

In ATL, Tax-mediated nuclear factor-κB (NF-κB) activation plays an important role in the proliferation of ATL cells and in the transforming activity of HTLV-1 (Yamaoka et al., 1996). Activation of NF-κB by Tax includes both the classical and alternative pathways. Contrary, HBZ selectively suppresses the p65 mediated classical NF-κB pathway without inhibiting alternative NF-κB signaling (Zhao et al., 2009). There are two mechanisms involved in this inhibition. One mechanism is that HBZ inhibits p65 DNA-binding ability via interacting with p65. Another is that HBZ increases the expression of PDLIM2, an E3 ubiquitin ligase for p65, leading to the increase of p65 ubiquitination and degradation (Zhao et al., 2009). Both activation and bZIP domains are necessary for the inhibition of classic NF-κB pathway by HBZ. The selective modulation of classical NF-κB pathway by HBZ might explain the predominant activation of alternative pathway, and perhaps to oncogenesis. PDLIM2 has been reported to suppress Tax-mediated tumorigenesis by targeting Tax into nuclear matrix for proteasomal degradation (Yan et al., 2009). Together, this may offer an explanation for the repression of Tax in ATL cells, by which HBZ activates the expression of PDLIM2 and PDLIM2 subsequently functions to degrade Tax. The updated report showed that HBZ inhibits the acetylation of p65. It may contribute to the repression of classical NF-κB pathway by HBZ.

Recent studies suggest that the classical NF-κB pathway is mostly involved in innate immunity and inflammatory responses. The inhibition of NF-κB pathway is common among different viruses, suggesting that these activities are important for viral survival and infection. HTLV-1 might facilitate escape from the host immune attack by suppressing the classical NF-κB pathway by HBZ.

### INDUCED TREG DIFFERENTIATION THROUGH ENHANCING TGF-β SIGNALING

Adult T-cell leukemia cells, present a CD4^+^CD25^+^ phenotype, have the same markers as those of natural regulatory T cells (Tregs; Matsuoka and Jeang, 2007). Expression of FoxP3 was detected in two-thirds of ATL cases, indicating that ATL may originates from natural Treg cells infected with HTLV-1 (Karube et al., 2004). Recently, we found that transgenic expression of HBZ increases Foxp3^+^ Treg cells and effector/memory T cells, leading to development of T-cell lymphomas and systemic inflammatory diseases (Satou et al., 2011). It is well known that TGF-β signaling is critical for the development of Treg. Our study showed that HBZ enhanced TGF-β signaling in a p300-dependent manner (Zhao et al., 2011). HBZ, Smad3, and p300 formed a ternary complex, and the association between Smad3 and p300 was enhanced in the presence of HBZ. HBZ could overcome the suppressive effect of Tax on TGF-β pathway. The enhancement of TGF-β signaling by HBZ leads to the up-regulation of Foxp3 in naïve T cells (Zhao et al., 2011). This might account for why HTLV-1 infection increases Tregs *in vivo*. Interestingly, HBZ maintains the cell growth even with the presence of TGF-β, and HBZ does not influence the expression of genes associated with cell cycle and proliferation. It indicates HBZ selectively modulates actions of TGF-β/Smad signaling pathway.

By enhancing TGF-β pathway and Foxp3 expression, HBZ enables HTLV-1 to convert infected T cells into Tregs, which is thought to be a critical strategy for virus persistence.

### PATHOGENIC FUNCTION OF HBZ

Tax is recognized as an oncoprotein, since it immortalizes primary T cells and confers proliferative properties to HTLV-1-infected cells. However, transcript of the *tax* gene is detected in only 40% of ATL cases. In Tax transgenic mice, cancers were induced depending on the promoter used (Lairmore et al., 2005). To better understand the pathogenic function of HBZ, we generated transgenic mice expressing HBZ under mouse CD4 promoter and enhancer. The HBZ transgenic mice (HBZ-Tg) present many similarities in symptoms and immunological features with HTLV-1-infected individuals (Satou et al., 2011). In the total splenocytes of HBZ transgenic mice, the percentage of CD4-positive T cells increased. In addition, HBZ promotes the proliferation of thymocytes from transgenic mice. More than one third of HBZ-Tg mice developed T-cell lymphomas after a long latent period. To study the underlying molecular mechanisms, we analyzed the phenotype and function of T cells and found CD4^+^Foxp3^+^ Tregs and effector/memory CD4^+^ T cells increased *in vivo*. The high percentage of Foxp3^+^ cells in HBZ-Tg mice is also observed in lymphoma tissues from human ATL patients. However, the increased CD4^+^Foxp3^+^ Treg cells in HBZ transgenic mice were functionally impaired since HBZ could physically interact with Foxp3 and NFAT, thereby impairing the suppressive function of Treg cells (Satou et al., 2011). In addition, most of HBZ transgenic mice spontaneously develop inflammatory lesions in the skin and lung, and CD3^+^CD4^+^ T cells infiltrated into the dermis and epidermis in the lesions. These findings are similar to those observed in HTLV-1 carriers.

### IMPAIRED CELL-MEDIATED IMMUNITY

Viruses have strategies to escape the host immune attack and to replicate *in vivo*. HTLV-1 infection is complicated by opportunistic infections. However, it was still unknown how HTLV-1 causes immune deficiency. Recently, we found that HBZ transgenic mice were more vulnerable to the infections of herpes simplex virus type 2 (HSV-2) and *Listeria monocytogenes* (LM) than non-Tg mice (Sugata et al., 2012). The production of Th1 cytokines, such as IFN-γ, IL-2, and TNF-α, was suppressed in HBZ-Tg mice. Moreover, the impaired production of IFN-γ is also observed in primary CD4 T cells from ATL patients. This study has shown that HBZ suppresses the IFN-γ promoter activity through suppressing NFAT and AP-1 signaling pathways (Sugata et al., 2012). Together, it is the first report shown that HBZ inhibits CD4 T-cell responses, resulting in impaired host immunity *in vivo*.

## THE RELATIONSHIP BETWEEN TAX AND HBZ

Accumulating evidence showed that Tax and HBZ are two important factors for ATL. The crossover and the interaction between these two proteins are important for the HTLV-1 oncogenesis. Tax could transform rodent cells and immortalizes human primary T cells, moreover, it enhances viral replication, supports cellular proliferation, inhibits apoptosis, impairs cell-cycle checkpoints, and induces DNA damages. Although Tax has pleiotropic functions as mentioned above, ~60% of all ATL cases did not express Tax. Moreover, abortive genetic changes, including deletions, insertions, and non-sense mutations, were frequent in all viral genes include the *tax* gene. Non-sense mutations in the *tax* gene were also observed in proviruses from asymptomatic carriers. Contrary to the *tax* gene, HBZ is consistently expressed in all ATL cells and remains intact through the whole period of ATL development. Tax plays a central role in the immortalization of T-lymphocytes in cell culture, on the contrary, HBZ was dispensable for viral-induced immortalization of primary human T cells (Arnold et al., 2006). Tax is the major target of cytotoxic T-lymphocytes (CTLs) *in vivo *(Kannagi et al., 1991), while HBZ is not a target antigen for HTLV-1-specific CTLs (Suemori et al., 2009). Since HBZ promote the T-cell proliferation and induced lymphoma and chronic inflammation which is similar to those in HTLV-1-infected individuals, we believe HBZ play more important role in the maintenance of HTLV-1-induced transformation.

Interestingly, Tax and HBZ have opposite effect on the regulation of cellular signaling pathways (**Table 1).** Tax activates 5′ LTR transcription; enhances AP-1 and classic NF-κB signaling, and suppresses TGF-β pathway. Contrary, HBZ suppressed Tax-mediated HTLV-1 viral transcription; inhibit activated AP-1 and classical NF-κB signaling. However, HBZ enhances TGF-β pathway. HTLV-1 may take advantage of this opposite function of Tax and HBZ on regulating signaling pathway to allowing the better survive of HTLV-1-infected cells and escape the immune surveillance.

**Table 1:** Character and regulatory function ofTax and HBZ.

Strain or plasmid	Tax	HBZ
Expression in ATL cells	~40%	100%
Contain genetic changes	Yes	No
Function as mRNA	ND	Yes
Immortalize T cells	Yes	No
Target by CTL	Yes	No
HTLV-1 5’LTR transcription	Activate	Inhibit
Classical NF-κB pathway	Activate	Inhibit
Alternative NF-κB pathway	Activate	No
AP-1 activity	Activate	Inhibit
AP-1 activity	Activate	Activate
T-cell proliferation	Activate	Activate
TGF-β signaling	Inhibit	Activate
TGF-β signaling	Enhance	Suppress

## ANTISENSE TRANSCRIPT OF RETROVIRUS

**FIGURE 3:**
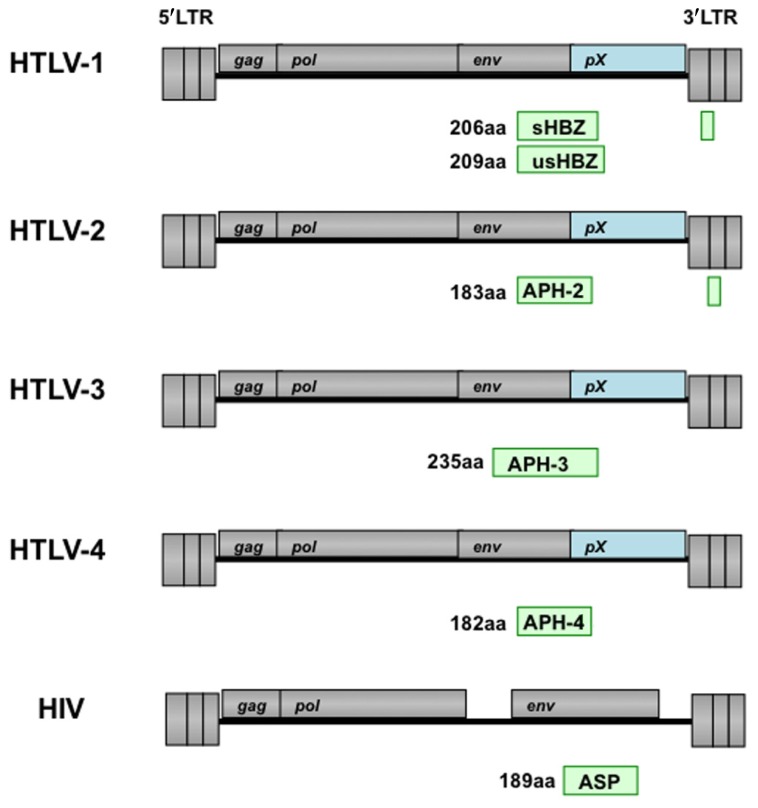
**Schematic representation of antisense transcripts in HTLV and HIV proviral genomes.** Position of antisense transcript encoded proteins (APH-2/3/4) in HTLV-2, HTLV-3, and HTLV-4 is similar as HBZ in HTLV-1. The antisense protein (ASP) encoded by the minus strand of HIV genome is indicated. The number indicated the total amino acids of each protein.

There are four kinds of HTLVs have been identified, and their oncogenic property is different. HTLV-1 mainly infected CD4^+^ T cells. However, HTLV-2 tends to target CD8^+^ T cells, and induces cancer only in rare cases. Unlike Tax1, Tax2 which encodes by HTLV-2 does not have PDZ domain-binding motif (PBM). Since PBM is important for the transforming activity, such differences between Tax1 and Tax2 may responsible for the different pathogenicities of HTLV-1 and HTLV-2 (Hirata et al., 2004). It was reported that HTLV-2 produced an antisense transcript termed antisense protein (ASP) of HTLV-2 (APH-2; **Figure 3;** Halin et al., 2009). Like the s*HBZ* gene, APH-2 mRNA is transcribed from the 3′ LTR, and spliced and polyadenylated. APH-2 does not contain bZIP domain but it still could interact with CREB and suppresses Tax2-dependent LTR activation. In addition, APH-2 did not colocalize with the nucleolus, although in non-T and T cells, it did have nuclear localization. Importantly, APH-2 was correlated with proviral load, while it does not promote cell proliferation and does not cause lymphocytosis.

Recent reports showed that HTLV-3 and HTLV-4 produce a spliced and polyadenylated antisense transcript (Barbeau and Mesnard, 2011). These two proteins were named as APH-3 and APH-4, respectively, since both proteins lack consensus bZIP domain (**Figure 3).** But, they retained the capacity to inhibit LTR activation that mediated by their respective Tax proteins. This study demonstrated a nuclear-restricted pattern for APH-4 while APH-3 was localized both in the cytoplasm and in the nucleus.

A naturally occurring antisense RNA was identified in the cell lines infected with HIV-1, and encodes an ASP with an apparent molecular mass of 19 kDa (**Figure 3;** Vanhee-Brossollet et al., 1995; Clerc et al., 2011). Antibodies against this protein have been detected in the sera of HIV-positive individuals. However, it remains to be elucidated the function of this ASP *in vivo*.

## PERSPECTIVES

Intensive studies on the leukemogenesis of ATL since the discovery of HTLV-1 have revealed some molecular mechanisms by which viral proteins induce viral replication and cellular transformation. However, the precise mechanisms of oncogenesis by HTLV-1 remain to be clarified. Recent progresses on the researches of the *HBZ* gene give new ideas into the mechanisms of oncogenesis. HBZ is the only viral gene that is expressed in all ATL cases.Except the *HBZ* gene, non-sense mutations of other viral genes are detected before integration of provirus. Importantly, HBZ maintains the cell survival when Tax is silenced. These data collectively indicate that HBZ protein has predominate function on the oncogenesis of HTLV-1. Further studies are needed to clarify the oncogenic function of HBZ and develop therapy targeted against HBZ based on the growing knowledge of HTLV-1 molecular biology.

## Conflict of Interest Statement

The authors declare that the research was conducted in the absence of any commercial or financial relationships that could be construed as a potential conflict of interest.
